# Predictors of Positive and Negative Risk-Taking in Adolescents and Young Adults: Similarities and Differences

**DOI:** 10.5964/ejop.2169

**Published:** 2021-02-26

**Authors:** Joanna Fryt, Monika Szczygiel

**Affiliations:** a Institute of Psychology, Pedagogical University of Krakow, Krakow, Poland; Edinburgh Napier University, Edinburgh, United Kingdom

**Keywords:** positive risk-taking, negative risk-taking, sensitivity to reward and punishment, tolerance to ambiguity, adolescence and young adulthood

## Abstract

Although the risk-taking can potentially result in positive and negative outcomes, most of the researchers focused on its negative, not positive manifestations. Recently, Duell and Steinberg proposed a framework that clarifies the features of positive risk-taking. Research comparing positive and negative risk-taking increased and new measures have been developed. The presented study was designed to examine how the construct of positive risk-taking differs or overlaps with its opposite, negative risk-taking, and whether both are predicted by the same or different factors. Two hundred fifty eight (258) adolescents and young adults (aged 16-29) participated in the study. We tested self-reported sensitivity to reward and punishment, self-control, tolerance to ambiguity, trait anxiety, and gender as possible predictors of positive and negative risk-taking. We also referred both types of risk-taking to domain-specific risk-taking. We found that positive risk-taking is driven by sensitivity to reward and tolerance to ambiguity, and occurs especially in the social domain. Negative risk-taking is driven by gender, sensitivity to reward and (low) sensitivity to punishment, and occurs in all domains except social. Results indicate that positive risk-taking is chosen for exploration and personal growth by people who look for rewards in the social world and is done in a socially accepted way. Negative risk-taking is chosen by people who are not discouraged by severe negative effects and look for rewards outside existing norms.

Risk-taking is considered not only as dangerous or illegal behavior like fast driving, binge drinking, or stealing but also self-challenging, socially accepted behavior like trying a new sport, standing by what you think is right, or performing in front of an unknown audience (Duell & Steinberg, 2019). According to the definition formulated in economics and decision science (Figner & Weber, 2011), all risk-taking can potentially result in positive and negative outcomes, and the greater the variability of what may happen, the greater the risk. Positive risk-taking is therefore not a behavior with only positive outcomes but a *desirable* risky behavior. It has been studied in various contexts under different, more or less overlapping terms: for example, *positive* risk-taking which is legal and not dangerous (Hansen & Breivik, 2001); *prosocial* risk-taking which is taking risk for others (Do, Guassi Moreira, & Telzer, 2017; Wood, Dawe, & Gullo, 2013); *adaptive* risk-taking which means a more advantageous choice (Blair, Moyett, Bato, DeRosse, & Karlsgodt, 2018; Humphreys, Lee, & Tottenham, 2013); *reasoned* risk-taking which is planned, not reactive (Maslowsky, Owotomo, Huntley, & Keating, 2019).

Recently Duell and Steinberg (2019) proposed a framework that clarifies the features of positive risk-taking: it benefits the individual's well-being (a person may gain something), its potential costs are mild in severity (there is no threat to health or safety) and it is socially acceptable. Conversely, negative risk-taking is considered as dangerous (with severe negative outcomes) or illegal. Probably due to the fact that risk-taking is a frequent cause of accidents which peaks during adolescence and emerging adulthood (Defoe, Dubas, Figner, & van Aken, 2015; Duell, Steinberg, Icenogle, Chein, & Chaudhary, 2018; Willoughby, Good, Adachi, & Tavernier, 2013), most of the researchers focused on its negative, not positive manifestations (van Duijvenvoorde, Blankenstein, Crone, & Figner, 2016). Research on positive risk-taking is, however, of great practical importance, given that there may be individual or contextual differences between young people taking positive and negative risk. In our study we examined how the construct of positive risk-taking proposed by Duell and Steinberg (2019) differs or overlaps with its opposite, negative risk-taking, and whether both are explained by the same or different factors.

The first question regarding positive versus negative risk-taking is whether they mainly differ in the severity of the outcomes or in the context (domain) in which the risk is taken. One way to answer this question is to refer positive and negative risk-taking to domain-specific risk-taking (Blais & Weber, 2006; van Duijvenvoorde et al., 2016), which means that if a person takes risk in one domain (e.g., drinking heavily at the party), he/she does not necessarily take it in another (e.g., sky diving). Although there is no research on this topic, it is possible that negative risk-taking, restricted to dangerous and illegal behavior, overlaps with risk-taking in the health/safety and ethical domains. Positive risk-taking may, in turn, overlap with risk-taking in social and recreational domains, in which a person may benefit his/her well-being (e.g., challenge herself, gain approval) in a socially acceptable way.

What *drives* positive and negative risk-taking is a more comprehensive question. First of all, there are strong indications that among factors predicting both types of risk-taking is sensation seeking or sensitivity to reward (SR). Sensation seeking was found to be a predictor of the taking of both legal and illegal risks (Hansen & Breivik, 2001), as well as those with and without negative health outcomes (Fischer & Smith, 2004). Reward drive (a part of Gray's Behavioral Activation System, associated with SR and approach motivation) predicted substance use, physical risk (sports), performance risk (singing, dancing) and prosocial behavior (Wood et al., 2013). In neuroscientific studies the activity of reward system is a well-known predictor of risk-taking (Shulman et al., 2016) and prosocial behavior (Do et al., 2017). As it was found that the activity of reward system benefits experience-based learning in adolescents (Peters & Crone, 2017), some authors consider it crucial for adaptive, exploratory risk-taking which usually peaks in this period of life (Romer, Reyna, & Satterthwaite, 2017).

Factors that predict only positive or negative risk-taking, or have contrary associations with them, are less recognized. As it was found that low sensitivity to punishment (SP) predicts more alcohol and marijuana use in people with poor inhibitory control (Kahn et al., 2018), it is possible that SP is a greater regulator of risk-taking with severe negative outcomes. A similar association may exist between such risk-taking and low trait anxiety (when a person is not afraid of severe outcomes). However, when we think of adolescents and young adults as sensitive to peers and oriented towards the social world (van Hoorn, Fuligni, Crone, & Galvan, 2016), we may not be sure whether they consider health or social outcomes as *severe.* Compared to adults, adolescents are more reactive to social threats like failure or rejection. It is interesting whether SP (or anxiety) prevents them from negative risk-taking (which threatens health and safety) or from positive risk-taking (which threatens status and well-being in a peer group).

Self-control (and related constructs) may have contrary associations with positive and negative risk-taking. It was found that deliberation (a part of consciousness construct) predicted risk-taking with but not without negative health outcomes (Fischer & Smith, 2004). Rash impulsiveness positively predicted substance use but negatively predicted performance risk and prosocial behavior (Wood et al., 2013). While there are strong indications that negative risk-taking is driven by impulsivity (Bjork & Pardini, 2015; Shulman et al., 2016), it is unclear whether positive risk-taking is accompanied by self-control or it requires it (Duell & Steinberg, 2019; Romer et al., 2017).

Tolerance to ambiguity was found to drive reckless and rebellious risk-taking (Blankenstein, Crone, van den Bos, & van Duijvenvoorde, 2016; van den Bos & Hertwig, 2017). Whether it drives positive risk-taking remains thus far unexplored. Authors interpreting the fact that adolescents are more tolerant to ambiguity than adults (Tymula et al., 2012) emphasize that it may be adaptive to enhance exploration and learning in an unfamiliar world (and the world for a teenager is more unfamiliar than for an adult). It is worth noting that tolerance to ambiguity is a construct related to openness to experience that was found to predict risk-taking in the social and recreational domains (Weller & Tikir, 2011). Authors of this study examined associations between all HEXACO personality traits and domain specific risk-taking. They found that while taking risk in social and recreational domains is associated with high openness to experience, taking risk in ethical and health/safety domains is associated with low honesty/humility.

Finally, women are seen less as risk takers than men (Duell et al., 2018; Harris & Jenkins, 2006). In decision science such difference is explained in terms of risk perception, as women, compared to men, perceive risk as higher in all domains except the social one (Figner & Weber, 2011; Harris & Jenkins, 2006). This suggests that gender may predict negative but not positive risk-taking, the potential costs and benefits of which are often of a social nature.

In our study we evaluated positive and negative risk-taking accordingly to the definition proposed by Duell and Steinberg (2019), in a group of adolescents and young adults (aged 16-29). We evaluated SR and SP, self-control, tolerance to ambiguity, and trait anxiety in order to examine which of them drive positive or negative risk-taking. In older participants we were able to refer positive and negative risk-taking to domain-specific risk-taking. We hypothesized that: 1) positive risk-taking is positively predicted by SR, self-control and tolerance to ambiguity; 2) negative risk-taking is positively predicted by SR and negatively predicted by SP, self-control and trait anxiety; also, that women take less negative risk than men; 3) positive risk-taking is predicted by risk-taking in the social and recreational domains; 4) negative risk-taking is predicted by risk-taking in the health/safety and ethical domains.

## Method

### Participants

Two hundred fifty eight (258) participants (123 women, 128 men, 7 no data) at the mean age of 20.55 (*SD* = 2.29, 16-29 range, mainly 18-22 years) took part in the study. The result of the Kolmogorov-Smirnov test (K-S = 0.13, *p* < .001) indicates that the age distribution slightly deviates from normal and is right-skewed. The sample contained both high school and university students with different academic profiles (e.g., mathematics, computer science, cognitive science, history, law, management), recruited during school meetings. All participants provided written informed consent; in the case of underage students parental consent was also obtained.

### Measures

#### Positive Risk-Taking Scale (PRTS)

We used a new scale of Duell and Steinberg (2020) to assess positive risk-taking. This contains 14 items describing behaviors that are examples of positive risk-taking (according to the authors' definition) and can be performed by a young person in social, academic or extracurricular contexts. In order to emphasize the riskiness of the described behaviors, items are constructed in a way that indicates the uncertainty of possible outcomes (e.g., “tried a new sport where you may have embarrassed yourself” instead of “played a sport”). Participants are asked whether and how many times during the last 6 months they have done the things described. Originally, the answers were assessed on a 4-point scale from 1 (*none*) to 4 (*more than five times*) but to be able to compare the measures of positive and negative risk-taking we changed the response format to a 5-point scale from 1 (*never*) to 5 (*very often*). Two indicators of positive risk-taking are proposed by the authors: frequency score (PRTS-F) – the arithmetic mean of response to all items, and variety score (PRTS-V) – the proportion of behaviors that have been performed at least once to all measured behaviors. In our study the Cronbach's α of a frequency score was .80, and for a variety score it was .71.

#### Risk Behavior Questionnaire (RBQ)

To assess negative risk-taking we used a questionnaire developed for the purpose of our previous studies. The measure is based on the Adolescent Risk-Taking Questionnaire (Gullone, Moore, Moss, & Boyd, 2000), but its items were modified to make it applicable to both adolescents and young adults. It contains 29 behaviors that meet the criteria of negative risk-taking (they may have severe negative outcomes or are illegal). Participants assess how often they perform the described actions using a 5-point scale from 1 (*never*) to 5 (*very often*). Analogically to the PRTS, we calculated two indicators of negative risk-taking: the mean response to all items is a frequency score (RBQ-F); the proportion of behaviors that have been performed at least once to all behaviors is a variety score (RBQ-V). The Cronbach's α of the former was .88, for the latter it was .87.

#### Domain-Specific Risk-Taking Scale (DOSPERT)

To assess specific risk-taking domains we used the scale of Blais and Weber (2006) in university students only as it is designed for adult populations.^1^1The tentative version of adolescent DOSPERT scale has been developed by Figner and Weber and its brief description can be found in van Duijvenvoorde et al. (2016). The scale consists of risky behaviors typical for adolescents 13-17 years old, originated from five life domains (same as in the adult version). The scale contains 30 risky behaviors originating from five life domains: ethical (D-E), financial (D-F), health/safety (D-H/S), social (D-S) and recreational (D-R). Participants are asked how likely it is that they would do the things described if they were to find themselves in those situations. The answers are assessed on a 7-point scale from 1 (*extremely unlikely*) to 7 (*extremely likely*). The mean response to all items in a given subscale is the measure of risk-taking in a given domain. The Cronbach's α of the general score was .84, for the ethical domain it was .65, for the financial domain .77, for the health/safety domain .63, for the social domain .62, and for the recreational domain .79.

#### Short Version of the Sensitivity to Punishment and Sensitivity to Reward Questionnaire (SPSRQ-SF)

We used the Cooper and Gomez (2008) questionnaire adapted by Wytykowska, Bialaszek, and Ostaszewski (2014) that measures sensitivity to Gray's BIS system and the BAS system with separate subscales. It contains 24 yes/no statements. The mean response to all items in the subscale was the measure of SP and, respectively, SR. The Cronbach's α of SP subscale was .82 and for the SR subscale it was .64.

#### Self-Knowledge New Sheet (NAS-50)

A self-control questionnaire created by Necka et al. (2016) was administered. It contains 50 items divided into five subscales: goal maintenance (GM), proactive control (PC), initiative and persistence (IP), switching and flexibility (SF), inhibition and adjournment (IA). Participants assess to what extent the statements described characterize them using a 5-point scale from 1 (*definitely not*) to 5 (*definitely yes*). The higher mean response to all items, the higher the self-control. The Cronbach's α of the general score was .86.

#### Tolerance to Ambiguity Scale (TAS)

A self-report scale of McQuarrie and Mick (1992) adapted by Czajeczny (2016) was used to assess tolerance to ambiguity. It contains 12 statements with which participants can agree to varying degrees. The answers are assessed on a 7-point scale from 1 (*definitely not agree*) to 7 (*definitely agree*). The mean response to all items was the measure of tolerance to ambiguity. The Cronbach's α of the scale was .64.

#### State-Trait Anxiety Inventory (STAI)

To measure trait anxiety, we used the adaptation of Spielberger, Strelau, Tysarczyk, and Wrzesniewski (2011). It contains 20 statements about how participants usually feel. The answers are assessed on a 4-point scale from 1 (*definitely no*) to 4 (*definitely yes*). The mean response to all items was the measure of trait anxiety. The reliability of the scale calculated by Cronbach's α was .88.

### Procedure

Participants completed a set of self-report questionnaires in groups. This took place during one meeting in their schools, lasting about 45 minutes. Participants were assured anonymity and the possibility of receiving feedback about their results. They were not rewarded for participation. As the measure of domain-specific risk-taking is designed for adult populations (Blais & Weber, 2006), it was not included in the test battery for high school students.

## Results

The analyses of the data were conducted using the IBM SPSS Statistics (Version 24). At first, we present descriptive statistics and results of Pearson's correlation coefficient which was the basis for selecting predictors to the linear regression models. We were interested in the predictions of positive and negative risk-taking and we measured these variables using two indicators: frequency and variety. Because the results for both indicators are quite consistent, we decided to include in the description only the frequency results (PRTS-F, BRQ-F) and show the variety results in the Appendix (PRTS-V, BRQ-V).

### Descriptive Statistics and Matrix Correlation

In Table 1 we present descriptive statistics and the results of Pearson's correlation coefficient for all variables that we used in the study.

**Table 1:** Descriptive Statistics and Matrix Correlation

Variable	*M*	*SD*	RBQ-F	SR	SP	NAS-50	TAS	STAI	D-E	D-F	D-R	D-H/S	D-S
PRTS-F	2.81	0.57	0.31***	0.31***	0.20*	0.07	0.31***	-0.12	0.24*	0.26**	0.18*	0.15	0.35***
RBQ-F	1.68	0.46		0.43***	-0.29***	-0.22*	0.14	-0.14	0.49***	0.41***	0.38***	0.55***	0.23**
SR	5.65	2.15			-0.26**	-0.06	0.05	-0.16*	0.28***	0.31***	0.12	0.23**	0.22**
SP	6.41	3.70				-0.35***	-0.24**	0.60***	0.03	-0.08	-0.29***	-0.11	-0.36***
NAS-50	3.28	0.40					0.08	-0.34***	-0.42***	-0.06	0.02	-0.32***	-0.03
TAS	3.57	0.68						-0.14	0.01	0.16*	0.24*	-0.06	0.44***
STAI	2.28	0.44							0.09	-0.15	-0.12	0.09	-0.19*
D-E	2.26	1.03								0.25**	0.11	0.46***	0.15*
D-F	2.73	1.15									0.26**	0.27**	0.08
D-R	3.91	1.42										0.43***	0.40***
D-H/S	3.72	1.17											0.23**
D-S	5.12	0.95											

*Note. N =* 165. PRTS-F = positive risk-taking; RBQ-F = negative risk-taking; SR = sensitivity to reward; SP = sensitivity to punishment; NAS-50 = self-control; TAS = tolerance to ambiguity; STAI = trait anxiety; D-E = risk taking in ethical domain; D-F = financial domain; D-H/S = health/safety domain; D-R = recreational domain; D-S = social domain.

*p < .05. **p < .01. ***p < .001.

Positive risk-taking moderately and positively correlates with SR (*r* = .31, *p* < .001) and tolerance to ambiguity (*r* = .31, *p* < .001) and weakly and negatively with SP (*r* = -.20, *p* < .05). There is no relationship between positive risk-taking, self-control and trait anxiety. Of the risk-taking domains, positive risk-taking correlates positively, weakly, or moderately, with ethical (*r* = .24, *p* < .05), financial (*r* = .26, *p* < .01), recreational (*r* = .18, *p* < .05), and social domains (*r* = .35, *p* < .001) but not with the health/safety domain.

Negative risk-taking, similar to positive risk-taking but more strongly, positively correlates with SR (*r* = .43, *p* < .001) and negatively with SP (*r* = .29, *p* < .001). In contrast to positive risk-taking, negative risk-taking correlates significantly with self-control (*r* = -.22, *p* < .05) and is not related to tolerance to ambiguity. The absence of a relationship between negative risk-taking and trait anxiety is observed. Negative risk-taking correlates positively, from weak to strong, with all risk domains (ethical *r* = .49, *p* < .001; financial *r* = .41, *p* < .001; recreational *r* = .38, *p* < .001; health/safety *r* = .55, *p* < .001; and social *r* = .23, *p* < .001).

In the next step, the gender differences were tested by an independent sample *t*-test (see Table 2). The assumption of group equivalence was violated in risk-taking domains, however, normality of distribution in subgroups and homogeneity of variance were good. Additionally, the effect size was calculated (Lenhard & Lenhard, 2016), which takes into account group size.

**Table 2:** Gender Differences

Variable	Women		Men		Group comparison
	*M* (*SD*)	*n*	*M* (*SD*)	*n*	Independent sample *t*-test
PRTS-F	2.81 (0.62)	123	2.77 (0.58)	128	*t*(249) = 0.47, *p* = .63, *d* = 0.07
RBQ-F	1.58 (0.38)	123	1.81 (0.49)	119	*t*(240) = -4.02, *p* < .001, *d* = 0.52
SR	5.31 (2.14)	123	6.49 (2.08)	128	*t*(249) = -4.44, *p* < .001, *d* = 0.56
SP	6.89 (3.47)	123	5.93 (3.72)	128	*t*(249) = 2.11, *p* = .04, *d* = 0.27
NAS-50	3.26 (0.41)	123	3.34 (0.39)	128	*t*(249) = -1.60, *p* = .11, *d* = 0.20
TAS	3.55 (0.74)	121	3.68 (0.75)	115	*t*(234) = -1.28, *p* = .20, *d* = 0.17
STAI	2.39 (0.42)	123	2.19 (0.45)	116	*t*(237) = 3.62, *p* < .001, *d* = 0.46
D-E	2.10 (0.94)	107	2.60 (1.09)	65	*t*(170) = -3.20, *p* < .01, *d* = 0.49
D-F	2.49 (1.05)	107	3.12 (1.37)	65	*t*(170) = -3.56, *p* < .001, *d* = 0.52
D-R	3.81 (1.36)	107	4.03 (1.49)	65	*t*(170) = -1.00, *p* = .32, *d* = 0.15
D-H/S	3.57 (1.15)	107	3.97 (1.16)	65	*t*(170) = -2.21, *p* < .05, *d* = 0.35
D-S	5.06 (1.00)	107	5.18 (0.94)	65	*t*(170) = -0.85, *p* = .40, *d* = 0.12

*Note.* PRTS-F = positive risk-taking; RBQ-F = negative risk-taking; SR = sensitivity to reward; SP = sensitivity to punishment; NAS-50 = self-control; TAS = tolerance to ambiguity; STAI = trait anxiety; D-E = risk taking in ethical domain; D-F = financial domain; D-H/S = health/safety domain; D-R = recreational domain; D-S = social domain.

The results indicate that men exceed women in negative risk-taking, SR, ethical, financial, and health/safety risk domains and the differences are small/medium. Women in comparison to men have higher level of SP (small difference) and trait anxiety (medium difference). No gender differences were observed in positive risk-taking, self-control, tolerance to ambiguity, recreational and social risk domains.

### Predictors of Positive and Negative Risk-Taking

To find out and compare predictors of positive and negative risk-taking, two models were tested including the same independent variables (gender, SR, SP, NAS-50, TAS, STAI) and one of the dependent variables (PRTS-F or RBQ-F). Of all the independent variables that were included in the study as potential predictors of risk-taking (positive or negative), only those that significantly correlated with the dependent variable were included in the linear regression models. Each time, those independent variables that turned out to be insignificant in the first step of analysis were removed (Model 1) and a new linear regression model with significant predictors was tested to better fit the model to the data (Model 2).

To answer the questions about predictions of positive risk-taking, the regression linear model including SR and SP and tolerance to ambiguity was tested (see Table 3).

**Table 3:** Estimation of Parameters of the Linear Model

Parameter	β	*SE*	Statistic			
			Estimate	*SE*	*t*	*p*
Model 1^a^						
Intercept			1.51	0.24	6.21	< .001
SP	-0.07	0.06	-0.01	0.01	-0.09	.28
SR	0.27	0.06	0.08	0.02	4.41	< .001
TAS	0.30	0.06	0.25	0.05	4.99	< .001
Model 2^b^						
Intercept			1.51	0.24	6.21	< .001
SR	0.29	0.06	0.08	0.02	4.91	< .001
TAS	0.32	0.06	0.26	0.05	5.45	< .001

*Note*. The dependent variable is positive risk-taking (PRTS-F). Tested predictors are sensitivity to punishment (SP), sensitivity to reward (SR), and tolerance to ambiguity (TAS).

^a^*R*^2^ = .19, *F*(3, 238) = 19.32, *p* < .001. ^b^*R*^2^ = .19, *F*(2, 239) = 28.36, *p* < .001.

The results indicate that the first hypothesis was confirmed partially. As expected, SR and tolerance to ambiguity predict positive risk-taking, although self-control does not explain it. Two predictors in a similar way predict positive risk-taking (SR β = 0.29; TAS β = 0.32) but they explain only 19% of its variation.

To determine the answer to the question about predictors of negative risk-taking, an analogical model with an additional gender and anxiety trait variable was tested (see Table 4).

**Table 4:** Estimation of Parameters of the Linear Model

Parameter	β	*SE*	Statistic			
			Estimate	*SE*	*t*	*p*
Model 1^a^						
Intercept			1.17	0.22	5.32	< .001
Gender	-0.12	0.06	-0.11	0.06	-1.95	.05
SP	-0.20	0.08	-0.02	0.01	-2.55	.01
SR	0.34	0.06	0.07	0.01	5.36	< .001
TAS	0.09	0.06	0.05	0.04	1.48	.14
STAI	0.05	0.07	0.05	0.07	0.71	.48
Model 2^b^						
Intercept			1.49	0.11	13.78	< .001
Gender	-0.14	0.06	-0.13	0.05	-2.37	.02
SR	0.33	0.06	0.07	0.01	5.31	< .001
SP	-0.17	0.06	-0.02	0.01	-2.88	< .01

*Note*. The dependent variable is negative risk-taking (RBQ-F). Tested predictors are gender, sensitivity to punishment (SP), sensitivity to reward (SR), tolerance to ambiguity (TAS), and trait anxiety (STAI).

^a^*R*^2^ = .21, *F*(5, 227) = 13.58, *p* < .001. ^b^*R*^2^ = .21, *F*(3, 238) = 22.11, *p* < .001.

The hypothesis about predictions of negative risk-taking was partially confirmed. In line with our expectations, gender, SR and SP are important when predicting negative risk-taking, but self-control and trait anxiety proved to be insignificant. SR positively and moderately (β = 0.35), SP weakly and negatively (β = -0.17), gender weakly and negatively (β = -0.14), predict negative risk-taking. The tested model explains, similarly to positive risk-taking, the 21% variation of the dependent variable.

In the next step of testing predictions of positive and negative risk-taking we checked its relation to domain-specific risk-taking. Firstly, we tested a model that consists of ethical, financial, recreational, and social domains as predictors of positive risk-taking (see Table 5), and then the model that included all domains as predictors of negative risk-taking (see Table 6).

**Table 5:** Estimation of Parameters of the Linear Model

Parameter	β	*SE*	Statistic					
			Estimate	*SE*	*t*	*p*
Model 1^a^						
Intercept			1.45	0.23	6.32	< .001
D-E	0.16	0.07	0.08	0.04	2.04	< .05
D-F	0.18	0.07	0.08	0.04	2.39	< .05
D-R	-0.02	0.08	-0.01	0.03	-0.30	.77
D-S	0.34	0.08	0.20	0.04	4.44	< .001
Model 2^b^						
Intercept			1.43	0.22	6.41	< .001
D-E	0.15	0.07	0.08	0.04	2.06	< .05
D-F	0.17	0.07	0.08	0.03	2.41	< .05
D-S	0.33	0.07	0.19	0.04	4.72	< .001

*Note*. The dependent variable is positive risk-taking (PRTS-F). Tested predictors are risk-taking in ethical (D-E), financial (D-F), recreational (D-R), and social (D-S) domains.

^a^*R*^2^ = .18, *F*(4, 172) = 10.45, *p* < .001. ^b^*R*^2^ = .18, *F*(3, 173) = 13.98, *p* < .001.

**Table 6:** Estimation of Parameters of the Linear Model

Parameter	β	*SE*	Statistic			
			Estimate	*SE*	*t*	*p*
Model 1^a^						
Intercept			0.42	0.16	2.68	< .01
D-E	0.29	0.07	0.13	0.03	4.28	< .001
D-F	0.22	0.06	0.09	0.02	3.60	< .001
D-S	0.06	0.06	0.03	0.03	0.86	.39
D-H/S	0.28	0.07	0.11	0.03	3.97	< .001
D-R	0.14	0.07	0.05	0.02	2.05	< .05
Model 2^b^						
Intercept			0.52	0.11	4.99	< .001
D-E	0.29	0.07	0.13	0.03	4.43	< .001
D-F	0.22	0.06	0.09	0.02	3.56	< .001
D-H/S	0.29	0.07	0.11	0.03	3.99	< .001
D-R	0.17	0.07	0.05	0.02	2.53	< .05

*Note*. The dependent variable is negative risk-taking (RBQ-F). Tested predictors are risk-taking in ethical (D-E), financial (D-F), social (D-S), health/safety (D-H/S), and recreational (D-R) domains.

^a^*R*^2^ = .44, *F*(5, 163) = 27.66, *p* < .001. ^b^*R*^2^ = .44, *F*(4, 164) = 34.44, *p* < .001.

Three predictors: ethical, financial, and social risk-taking explain the variation in positive risk-taking of 18%. The results are partially in accordance with our assumptions. Social risk-taking is the strongest predictor of positive risk-taking (β = 0.33), while ethical (β = 0.15) and financial (β = 0.17) predictors are significant but definitely less important.

In accordance with our expectations, negative risk-taking is moderately predicted by risk-taking in health/safety (β = 0.29) and ethical (β = 0.29) domains. We did not assume that financial (β = 0.22) and recreational domains (β = 0.17) may be important, though, they are slightly weaker predictors. Compared to positive risk-taking, the magnitude of the explained variance of the dependent variable by these three variables is relatively large and amounts to 44%.

Summarizing the predictions of positive and negative risk-taking, it should be noted that both are predicted by SR (although slightly stronger in the latter) and that positive risk-taking is predicted by tolerance to ambiguity whereas negative risk-taking is predicted by gender and SP. The similarity between these two kinds of risk-taking is visible when referring them to domain-specific risk-taking: ethical and financial, but the differences are revealed in the social domain – characteristic for positive risk-taking, and the health/safety and recreational domains – specific for negative risk-taking.

## Discussion

The results of our study indicate that positive and negative risk-taking share some common background and differ in certain behavioral patterns. Positive risk-taking, driven by SR and tolerance to ambiguity, occurs mainly in the social domain. Such a finding suggests that it is chosen for exploration and personal growth, and that people who take positive risks look for rewards in the social world, in a socially accepted way. On the other hand, negative risk-taking, driven by SR, (low) SP, and gender takes place in ethical and health/safety, and, to a lesser extent, in financial and recreational domains. This suggest that it is chosen by people who look for rewards outside the existing norms and are not discouraged by severe negative outcomes. The finding that women compared to men take less negative (but not positive) risk is compatible with the explanation that they perceive risk as higher in all domains except the social one (Harris & Jenkins, 2006).

Our findings may be firstly considered in the light of associations between domain-specific risk-taking and HEXACO personality traits (Weller & Tikir, 2011). People high in openness to experience take risk in the social and recreational domains, and they highly value the benefits of risk-taking in these domains. In turn, people low in honesty/humility, associated with reluctance towards norms and cooperation, take risk in the ethical and health/safety domains. These findings support the interpretation that exploration and running contrary to norms are important drivers for positive and negative risk-taking.

While linking negative risk-taking with reluctance towards norms and low fear of danger does not raise doubts, one may ask why exploration would drive only positive risk-taking. When we look at the examples of negative risk-taking, we note that some of them lack of the element of discovery (e.g., bullying, damaging property). However, many dangerous or illegal behaviors offer space for exploration (e.g., using drugs), especially if people experiment with them.

One possibility why people tolerant to ambiguity do not score high on negative risk-taking (but see Blankenstein et al., 2016; Van den Bos & Hertwig, 2017; who assessed tolerance to ambiguity by task) is that they explore both positive and negative risk-taking but they do not *repeat* the negative one. This interpretation finds support in neuroscientific research that distinguishes between exploratory and impulsive risk-taking (Bjork & Pardini, 2015; Romer et al., 2017). Exploratory risk-taking increases in adolescence with sensation seeking, self-control and experience-based learning; impulsive risk-taking results from poor self-control. Consequently, any risk-taking can be exploratory if only a person *learns* from the outcomes experienced (e.g., an adolescent experiments with drugs but does not become a habitual user).

We can also consider the possibility that it is social or academic aspirations that incline people who are tolerant to ambiguity to positive risk-taking. First, when we look at the examples of positive risk-taking, we note that many of them are associated with social or academic success (e.g., standing for a leadership position, taking a challenging course). Second, aspirations and cognitive capacity are known to increase exploration as opposed to exploitation (Mehlhorn et al., 2015). The essence of exploration, defined as constant switching between options, is to experiment with new alternatives; the essence of exploitation is to remain at one option. Those who choose to explore, gain information about their environment (“maximizers”); those who choose to exploit gain tangible rewards (“satisficers”) (Parker, Bruine de Bruin, & Fischhoff, 2007). To examine possible associations between academic or social aspirations and positive risk-taking, research cannot be limited to groups with specific aspirations (e.g., university students).

Finally, it is worth considering our results regarding self-control. Despite the supposition that it may have contrary associations to positive and negative risk-taking, we did not find it among the predictors. Interestingly, it was tolerance to ambiguity, not self-control that drives positive risk-taking. Such a result suggests that the behaviors proposed by Duell and Steinberg (2018) involve motivation for exploration and personal growth more than self-control. The lack of association between negative risk-taking and self-control may, in turn, indicate that it only occurs in a subgroup of people with poor control abilities (Bjork & Pardini, 2015).

In the study we tested predictions of positive and negative risk-taking focusing on their similarities and differences. Although we identified several factors that drive both or only positive or negative risk-taking in adolescents and young adults, the results are preliminary and conclusions should be drawn with caution. Future research should expand on younger adolescents and young adults who are not university students, firstly, considering that social or academic aspirations can increase positive risk-taking. Also, it is worth examining more thoroughly what behaviors are part of positive risk-taking for people from different age groups and backgrounds. This may include examining narratives of positive and negative risk-taking. It is possible that examples of positive risk-taking change from adolescence to adulthood and not considering what is typical and available at certain age may obscure potential age differences. Also, if social and academic domains predominate among examples of positive risk-taking, we can overestimate the role of social or academic competence in choosing positive risks. Certainly, the knowledge about what determines the choice of specific types of risk-taking is of great importance because it allows us to design environments that protect young people from severe negative effects.

## Appendix

The results of correlation and linear regression analysis including *variety* indicator of positive (PRTS-V) and negative risk taking (RBQ-V).

**Table A1:** PRTS-V and RBQ-V and Their Correlates

Variable	*M*	*SD*	RBQ-V	SR	SP	NAS-50	TAS	STAI	D-E	D-F	D-R	D-H/S	D-S
PRTS-V	0.77	0.16	0.23**	0.22*	-0.09	0.10	0.23**	-0.08	0.17*	0.25**	0.09	0.05	0.17*
RBQ-V	0.36	0.21	-	0.44***	-0.23**	-0.18*	0.14	-0.13	0.46***	0.42***	0.35***	0.48***	0.18*

*Note. N =* 165. PRTS-V = positive risk-taking; RBQ-V = negative risk-taking; SR = sensitivity to reward; SP = sensitivity to punishment; NAS-50 = self-control; TAS = tolerance to ambiguity; STAI = trait anxiety; D-E = risk taking ethical domain; D-F = financial domain; D-H/S = health/safety domain; D-R = recreational domain; D-S = social domain.

**p* < .05. ***p* < .01. ****p* < .001.

**Table A2:** Gender Differences in PRTS-V and RBQ-V

Variable	Women		Men		Group comparison
	*M* (*SD*)	*n*	*M* (*SD*)	*n*	Independent sample *t-*test
PRTS-V	0.75 (0.16)	123	0.76 (0.16)	128	*t*(249) = -0.15, *p* = .88, *d* = 0.06
RBQ-V	0.31 (0.18)	123	0.43 (0.22)	119	*t*(240) = -4.61, *p* < .001, *d* = 0.60

*Note.* PRTS-V = positive risk taking; RBQ-V = negative risk-taking.

**Table A3:** Estimation of Parameters of the Linear Model

Parameter	β	*SE*	Statistic			
			Estimate	*SE*	*t*	*p*
Model 1^a^						
Intercept			0.44	0.06	7.54	< .001
SR	0.22	0.06	0.02	0.01	3.66	< .001
TAS	0.24	0.06	0.06	0.01	3.98	< .001

*Note*. The dependent variable PRTS-V = positive risk taking; SR = sensitivity to reward; TAS = tolerance to ambiguity.

^a^*R*^2^ = .11, *F*(2, 239) = 15.40, *p* < .001.

**Table A4:** Estimation of Parameters of the Linear Model

Parameter	β	*SE*	Statistic			
			Estimate	*SE*	*t*	*p*
Model 1^a^						
Intercept			0.15	0.10	1.48	.14
Gender	-0.17	0.06	-0.07	0.03	-2.73	< .01
SP	-0.14	0.08	-0.01	0.01	-1.80	.07
SR	0.33	0.06	0.03	0.01	5.29	< .001
TAS	0.10	0.06	0.03	0.02	1.58	.12
STAI	0.02	0.07	0.01	0.03	0.32	.75
Model 2^b^						
Intercept			0.28	0.05	5.64	< .001
Gender	-0.18	0.06	-0.07	0.02	-3.00	< .01
SR	0.33	0.06	0.03	0.01	5.39	< .001
SP	-0.14	0.06	-0.01	0.01	-2.42	< .05

*Note*. The dependent variable RBQ-V = negative risk-taking; SP = sensitivity to punishment; SR = sensitivity to reward; TAS = tolerance to ambiguity; STAI = trait anxiety.

^a^*R*^2^ = .21, *F*(5, 227) = 13.61, *p* < .001. ^b^*R*^2^ = .22, *F*(3, 238) = 23.09, *p* < .001.

**Table A5:** Estimation of Parameters of the Linear Model

Parameter	β	*SE*	Statistic			
			Estimate	*SE*	*t*	*p*
Model 1^a^						
Intercept			0.54	0.07	8.07	< .001
D-E	0.10	0.08	0.02	0.01	1.35	.18
D-F	0.21	0.07	0.03	0.01	2.79	< .05
D-S	0.14	0.07	0.02	0.01	1.92	.06
Model 2^b^						
Intercept			0.55	0.07	8.33	< .001
D-F	0.23	0.07	0.03	0.01	3.23	< .05
D-S	0.16	0.07	0.03	0.01	2.16	< .05

*Note*. The dependent variable PRTS-V = positive risk-taking; D-E = risk-taking in ethical domain; D-F = financial domain; D-S = social domain.

^a^*R*^2^ = .08, *F*(3, 173) = 6.03, *p* < .001. ^b^*R*^2^ = .07, *F*(2, 174) = 8.09, *p* < .01.

**Table A6:** Estimation of Parameters of the Linear Model

Parameter	β	*SE*	Statistic			
			Estimate	*SE*	*t*	*p*
Model 1^a^						
Intercept			-0.14	0.08	-1.79	.08
D-E	0.29	0.07	0.06	0.01	4.07	< .001
D-F	0.23	0.06	0.04	0.01	3.60	< .001
D-S	0.01	0.07	0.01	0.01	0.15	.88
D-H/S	0.22	0.08	0.04	0.01	2.93	< .05
D-R	0.16	0.07	0.02	0.01	2.12	< .05
Model 2^b^						
Intercept			-0.13	0.06	-2.52	< .05
D-E	0.29	0.07	0.06	0.01	4.14	< .001
D-F	0.23	0.07	0.04	0.01	3.60	< .01
D-H/S	0.22	0.07	0.04	0.01	2.95	< .01
D-R	0.16	0.07	0.02	0.01	2.34	< .05

*Note*. The dependent variable RBQ-V = negative risk-taking; D-E = risk-taking in ethical domain; D-F = financial domain; D-S = social domain; D-H/S = health/safety domain; D-R = recreational domain.

^a^*R*^2^ = .38, *F*(5, 163) = 21.34, *p* < .001. ^b^*R*^2^ = .38, *F*(4, 164) = 26.83, *p* < .001.
